# Is walking netball an effective, acceptable and feasible method to increase physical activity and improve health in middle- to older age women?: A RE-AIM evaluation

**DOI:** 10.1186/s12966-021-01204-w

**Published:** 2021-10-19

**Authors:** F. E. Kinnafick, A. J. Brinkley, S. J. Bailey, E. J. Adams

**Affiliations:** 1 National Centre for Sport and Exercise Medicine, School of Sport, Exercise and Health Sciences, Loughborough University, Loughborough, LE11 3TU UK; 2 School of Sport, Rehabilitation and Exercise Sciences , University of Essex, Essex, CO4 3SQ UK; 3 School of Health Sciences, University of Nottingham, Nottingham, NG7 2UH UK

**Keywords:** Aging, Exercise, Intervention, Longitudinal, Physical activity, Wellbeing

## Abstract

**Background:**

Physical activity is a modifiable risk factor for health and wellbeing, all-cause mortality and healthy aging. However, for middle- to older-age females less is known about the benefits of *sports* participation on these outcomes. Further, the acceptability and feasibility of setting-up, implementing and maintaining sports-based programmes for an aging population is an understudied area of inquiry. The current study used the RE-AIM framework to investigate a nationwide Walking Netball (WN) programme.

**Methods:**

The evaluation used a mixed-methods approach incorporating a multiple-baseline study, quasi-experimental study, programme monitoring data and qualitative studies to evaluate the programme in the Women’s Institutes (WI) in England. Data were analysed using multilevel growth modelling, mixed-design ANOVAs, multilevel regression, t-testing, and thematic analysis. Data were triangulated to address each dimension of the RE-AIM framework.

**Findings:**

The programme reached 1.4% (*n* = 3148) of the WI population across 82.0% of WI regions in England and attracted inactive members at risk of ill-health. WN contributed to adaptations in physical function, mental health and wellbeing, social isolation, quality of life and increased physical activity. The adoption of the programme was successful with 87.7% WN groups’ maintaining participation beyond the 20-session initial delivery phase. Adoption was effective because of its set-up, peer-mentorship and long-term delivery; these factors likewise shaped implementation. Adapting and tailoring WN to the varying characteristics of participants within the WI and the facilities available, along with training delivery staff and providing resources are key programme components. The Walking Netball programme can be maintained through promotion within the local community, sustainable funding, inter-WI competitions, festivals and networks, multiple-hosts and continued host development.

**Conclusions:**

WN was found to be an acceptable, feasible and effective intervention to increase physical activity and improve health in middle- to older- aged women. Future programmes may consider adapted styles of set-up and delivery. These include adapting to people, places and spaces through personalised support and providing a range of resources. Future designs may seek to understand how participation can contribute to healthy aging through longitudinal research beyond 12-months.

**Study registration:**

The evaluation protocol was published in Open Science Framework in December 2018 prior to follow-up data collection being collected (https://www.osf.io/dcekz). Date of registration: 17 December 2018.

**Supplementary Information:**

The online version contains supplementary material available at 10.1186/s12966-021-01204-w.

## Background

An inverse relationship exists between physical activity (PA) participation and age [1]. Age and physical inactivity are risk-factors for non-communicable diseases (NCDs) [2] and physical decline in postmenopausal women [3]. NCDs and physical decline predict a reduction in quality of life, poor mental health, premature retirement, disability, social isolation, and reduced PA [3–8]. This underscores the importance of offsetting the declines in PA, physical function, quality of life, and mental health and illbeing that accompanies aging [4, 5]. PA can improve health and quality of life and contribute to healthy aging in middle- to older-age adults [4, 5, 9–12]. Increasingly, sports-based interventions are being adopted to promote health and increase PA within an aging population.

There is a paucity of evidence regarding the acceptability, feasibility and effectiveness of these types of interventions [13]. In working-age adults and masters athletes (> 35 years of age), sport is played at high intensity and requires complex motor patterns, balance, coordination and mobility [14]. However, high intensity sport with intermittent periods of active recovery may increase the risk-ratio for cardiac events and the complex changes in movement and eccentric muscle contractions may contribute to increased muscle soreness or injury, and low treatment acceptability in older-adults [14]. Walking sport minimises these risks while maintaining factors which contribute to long-term participation (e.g., team cohesion, competition, skill development) [14]. Previous research using walking sport has indicated participation in the short-, medium-, and long-term can improve clinical markers of musculoskeletal function, such as bone mineral content and density [15], osteogenic indicators [16]; predictors of physical function, such as mobility and muscular strength [17–19]; indicators of cardiorespiratory fitness and body composition [17, 19–21], and factors predictive of good mental health (e.g., identity and belonging, subjective wellbeing) [22, 23], quality of life [18] and healthy aging [18, 24–27].

Research on sports-based interventions has focused on soccer or adapted-games and sampled within small homogenous groups [13]. To compare effectiveness between sports, the array of factors which influence set-up, implementation, participation and sustainability must be considered [13]. In particular, there is a lack of research examining walking sport interventions designed for middle- to older-age postmenopausal women [13]. Whilst there are similarities in some environmental determinants of behaviour and factors which predict acceptability and feasibility [14], postmenopausal women differ to the middle- to older-age male and mixed-gender populations sampled in previous research both in terms of participation in PA and in terms of the physiological, musculoskeletal and psychological mechanisms which underpin health adaptations [28]. Further, while data from previous research is often drawn from randomised control trials, efficacy alone does not provide stakeholders with information regarding the extent to which an intervention needs to be adapted for a given setting, the target population, the context it is conducted within or the extent in which outcomes may be reproduced in similar groups [29]. Sports-based interventions are complex with a multi-componentry of interacting intrapersonal, interpersonal and environmental factors shaping set-up and participation [13]. Process evaluations understanding what, why, how and where an intervention works and the context in which it is delivered can inform both research and insight and shape the practise and policies of delivery-level stakeholders [30].

Process evaluations within sports-based health interventions for an aging population are sparse. However, systematic reviews do provide some insight into the factors which contribute to the design, implementation and maintenance of sport for middle- to older-age adults. More specifically, Jenkin and colleagues [14] found sports participation in older adults to be determined by health status, social community, sporting history, demographics, competition and organisation. These determinants of behaviour create enablers and barriers on the individual-, group-, and stakeholder-level. Additionally, Luo and colleagues [13] found moderately reported dropout rates in previous research and sparse data representing engagement in previous experimental interventions using soccer to promote health. This paucity of evidence representing acceptability and feasibility provides limited insight for stakeholders adopting sports-based interventions within an aging population. Given the emphasis placed on promoting and maintaining sports participation in government-level policy such as Sporting Futures [31], and strategies adopted by arms-length government agencies understanding the process which underpins participation in walking sport remains particularly important [32].

A range of process evaluation approaches exist [30]. Examining the reach, effectiveness, adoption, implementation, and maintenance of an intervention, the RE-AIM framework [33, 34] is a robust framework adopted within PA research. Reach represents the total quantity, demographics and representation of participants who are willing to engage in an intervention. Effectiveness relates to the impact of the intervention, both in terms of quantitative change but also subjective qualitative experiences and narratives. Adoption is the number, proportion and representation of settings (e.g., regions) and stakeholders (e.g., community groups) who are willing to initiate an intervention. Implementation covers the intervention’s fidelity, consistency of delivery, adaptions and degree of pragmatism. Finally, maintenance refers to the degree in which an intervention becomes integrated within an organisation’s routine practise, policies and activities.

The current study sought to address limitations of previous research in terms of sports studied, sampling and evaluation, and is an evaluation of the Walking Netball (WN) programme in England. Netball is a global sport played with seven players who are governed by rules regarding foot placement, positions and movement, move a ball around a court through passing, with the objective of shooting the ball through a raised net. At an unrestricted pace, netball is a sport played at high intensity and is associated with complex motor patterns, balance, coordination, mobility and attentional focus. WN has the capacity to address challenges to participation, associated with the fast-paced nature of the sport, by modifying rules requiring participants to walk rather than run. The current programme was designed with, and is delivered to, the Women’s Institute (WI) across England. The WI (www.thewi.org.uk) is a community organisation of an estimated 220,000 members, 6300 WI groups and 56 regional federations. The purpose of the WI is to provide opportunity, education and community to women through daily, weekly and/or monthly meetings, events and activities. Membership of the WI, whilst open to women of beyond the age of 18 years, is typically associated with middle- to older-age women. The aim of the WN programme was to encourage WI members to participate in netball; increase PA; promote mental and psychosocial health; and improve physical function and quality of life. The programme was delivered to the WI by England Netball and was funded by Sport England. The current study aimed to independently evaluate the WN programme via the RE-AIM framework [34]. Our objectives were to investigate the (i) reach, (ii) effectiveness, (iii) adoption, (iv) implementation, and (v) maintenance of the WN programme.

## Methodology

### The Walking Netball programme

The WN programme was designed by England Netball in collaboration with Age UK in 2017. The national programme began in 2018 and was advertised through articles, posters, social media, TV segments, WI press releases, meetings and conferences, and talks at WI meetings. The WN programme followed a sequential style to implementation whereby clusters of WIs began the programme in seven phases between March 2018–January 2020. Participation in the programme began with a WI or multiple WIs forming a WN group. Once initiated, weekly to fortnightly sessions of WN began. Each session had between 45 min to 1 h of practical delivery. Sessions took place in indoor settings (e.g., sport halls), outdoor courts and adapted spaces (e.g., church halls). They varied between WI groups but typically consisted of three components including (i) a warm-up activity, (ii) mini-games and drills, and (iii) a full game on a standard size court (30.5 × 15.3 m) or series of shortened games. The first 20 WN sessions were led by a WN *‘host’* employed by England Netball. The role of a WN host was not only to lead netball, but identify, recruit, mentor and train WI members to become WI *hosts* within the group. Following a process of supervision, mentorship and host training courses delivered by England Netball staff, WI hosts continued to lead WN activities. To support WI hosts, England Netball resources such as workshops, handout booklets, videos and access to social media groups were available centrally and regionally. In March 2020, the UK entered a series of nationwide and regional-level social-distancing measures in response to the COVID-19 pandemic. These restrictions caused WN to be suspended or continued with restrictions.

### RE-AIM evaluation

The WN programme was evaluated using mixed-methods and the RE-AIM framework [34]. Mixed methods data and interpretation through triangulation provided a robust analysis which is essential to the evaluation of complex interventions [29]. An overview of the evaluation and incorporation of the RE-AIM framework is provided in Table 1. Ethical approval from the project was granted by a UK based University Ethical Advisory Panel (Ref: R18-P044). The study conforms to, and was conducted in accordance with, the Declaration of Helsinki. All participants provided written informed consent. The methodology employed to address each RE-AIM dimension is detailed in the following sub-sections.

**Table 1:** Walking Netball RE-AIM overview

RE-AIM Dimensions	***Cross-sectional health survey (pre-baseline)***	***Multiple-baseline study (baseline to 12-months)***	***Programme data (monitoring systems, registers)***	***Quasi-experimental study (physical function)***	***Interviews and focus groups with WI members***	***Focus groups with WN hosts***	***Interviews with WI Hosts***
***Reach***	**X**		**X**		**X**	**X**	
***Effectiveness***		**X**		**X**	**X**	**X**	**X**
***Adoption***					**X**	**X**	**X**
***Implementation***		**X**	**X**		**X**	**X**	**X**
***Maintenance***			**X**		**X**		**X**

#### Multiple baseline study (reach, effectiveness, implementation)

##### Design

A multiple-baseline survey study [35] with five-time points and six-clusters was implemented in Spring 2018 to examine the reach, implementation and effectiveness of WN. A multiple-baseline design was preferable to a randomised cluster control trial due to stakeholder concerns regarding the exclusion of participants through the adoption of a control group. Multiple-baseline designs are effective in the evaluation of complex community-based health promotion programmes while the cross-sectional pre-baseline measures used provided assessments suitable for understanding reach [35, 36].

To prevent contamination (e.g., participants attending more than one WN session in their region), participants were recruited within six group level clusters. WI groups from across England formed study clusters. Each cluster was constructed from 10 WI’s, with each WI group containing an estimated 15 members (150 participants per cluster). Within multiple-baseline designs each cluster acts as its own control group, therefore eliminating the need for a traditional control group [35, 36]. Measures were taken on a rolling basis from March 2018 to September 2020. More specifically, all members of the WI (regardless of future participation in WN) were invited to participate in a pre-baseline survey. Upon registering for the WN programme, members completed a baseline-control survey and a series of follow-up surveys (3-, 6-, 12-months) throughout the WN programme.

##### Sampling

WI members were sampled through a total population method. The pre-baseline survey was promoted via WI communications, social media and by committee chairs at WI meetings. WI members were recruited into the study and subsequent baseline and follow up measures through an expression of interest in WN by the WI. Whilst not prevented from participation in WN, participants were excluded from the multiple-baseline study if they were (i) under 45 years old, (ii) not a member of the WI, or (iii) unable to provide informed consent. Surveys were completed by 859 WI members (pre-baseline measure), *n =* 828 (baseline measure), *n =* 308 (3-month follow up) (37.0% response rate), *n =* 226 (6-month follow up) (27.3% response rate), and *n =* 158 (12-month follow up) (19.1% response rate).

##### Measures

Data collected from participants included age, ethnicity, disability status, education, household income, marital status, WI group and living status. Further measures of PA behaviour (IPAQ-short form [37]), mental health and wellbeing (Warwick-Edinburgh Mental Wellbeing Scale [38]), loneliness and social isolation (UCLA loneliness scale V3 [39]), and quality of life (Dartmouth COOP Functional Assessment Chart [40]) were recorded. For a detailed overview of measures please see Additional File 1.

#### Programme monitoring data (reach, adoption, implementation)

Programme monitoring data was collected by England Netball and shared with the research team. Bespoke software monitored the number of programmes initiated and sustained (beyond 20 sessions of England Netball delivery), and the number of WI members beginning participation and maintaining participation beyond 20 sessions. Data representing the number of hosts trained and social media participation within the programme (e.g., YouTube ‘hits’ on virtual WN) was also recorded.

#### Physical function quasi-experimental study (effectiveness)

##### Design

A sub-sample of WI members participated in a 6-month quasi-experimental study. Groups of WI’s were assigned to either a WN or a control group. The control group was placed on a 6-month waiting list for WN and continued with their normal day-to-day lives. Quasi-experimental designs within health promotion evaluations are acceptable, pragmatic and feasible [36]. Baseline (T^0^) and follow-up (T^1^) measurement observations were undertaken at WI meetings. The schematic study design, recruitment strategy and attrition rate are presented in Additional File 2. Participants completed 26 sessions of WN. Over 6-months, participants on average attended 67.9% of WN sessions.

##### Sampling and participants

Participants were sampled through criterion-based sampling whereby participants were excluded on age (< 45 years); menopause status (premenopausal); unable to participate in PA or WN for 6-months, or unable to provide informed consent. Participants undertook a health screen and were excluded from the study if they reported health complications that would affect PA participation. Thirty participants in the WN group and 22 participants in the control group provided data at T^0^ and T^1^ (*n* = 8 participants dropped out of the control group over 6-months). The age range of the intervention group was 53 years to 77 years (mean 64.64 ± 6.31 years). Ages of participants in the control group ranged from 61 to 80 years (69.05 ± 4.16 years). An independent samples t-test indicated a difference of 2.41 years was not statistically meaningful between groups (t^(46)^=1.46, *p* = .149). The body mass index (calculated on self-reported height and weight) was 25.99 ± 6.81 kg/m^2^ in the WN group and 25.65 ± 6.81 kg/m^2^ in the control group at T^0^. Self-reported PA (mean minutes per-week) was 147.40 ± 92.02 min in the WN group and 179.00 ± 178.42 min in the control group. Intergroup differences in body mass index (BMI) (t^(38)^=.188, *p* = .852) and PA behaviour (t^(49)^=.189, *p* = .496) were not statistically different. All participants were retired or transitioning from full-time employment.

##### Measures

Participants completed measures of extremity and gait function (The Short Physical Performance Battery ‘SPPB’ [41]), functional movement (timed-up-and-go ‘TUG’ [42]), muscular strength (Takei; Digital 5401 [43]), and physical fitness (Six-Minute Walk Test ‘6MWT’ [44]). Further, details of the measures are provided in Additional File 3.

#### Qualitative data collection (interviews and focus group) (all RE-AIM dimensions)

Telephone interviews (*n* = 7) and face-to-face focus groups (*n* = 3; 31 WN participants) were conducted with WI members who were participating in WN and with those who had dropped out of the programme (e.g., due to injury). In addition, telephone interviews (*n* = 7) were conducted with WI hosts and three focus groups (*n* = 18 participants) were carried out with WN hosts. The purpose of these exploratory methods was to understand the participant experiences which represented each RE-AIM dimension. In all cases, participants were recruited via purposeful sampling through contacts within the WI and through representatives working with the WI and England Netball. Telephone interviews were adopted over in-situ conversations due to pragmatic reasons (e.g., logistics within the population and geographical spread of participants). Interviews and focus groups were conducted with participants from London, the Midlands, and the South, North, West and East of England. Interviews and focus groups were conducted using a semi-structured guide informed by literature (available on request). Questions covered the reach, effectiveness, adoption, implementation and maintenance of the programme. On average interviews lasted 61 min while focus groups lasted 90 min. The average age of WI members was 64 years, while the average age of WN hosts was 37 years. Participants represented a range of backgrounds, experiences and years playing or leading netball.

### Qualitative data analysis

Data collected from interviews and focus groups was transcribed verbatim and a thematic analysis [45] was undertaken using NVivo Version 12. Our analysis used inductive and deductive reasoning to investigate commonality in the data. Following a process of coding, data was segregated and grouped into themes (e.g., enablers to participation) and sub-themes (e.g., social support). Analysis was conducted independently for each study (e.g., focus groups with WN hosts) and then triangulated [46] as a broader analysis to address each dimension within the RE-AIM framework. An overview of the themes and sub-theme identified during this analysis is provided in Additional File 4. Members of the research team gave their consensus on the data and identified themes.

### Quantitative data analysis

Data analysis was conducted using MLwiN Version 3.04 for multilevel models and JASP Version 0.10.2 for general linear models. Descriptive statistics (mean ± standard deviation) were calculated for continuous variables and frequencies for categorical variables. All variables were normally distributed (±1.96) and met the assumptions required for the respective statistical analysis. Multilevel modelling was used in two-forms to address reach and effectiveness. More specifically, to examine the demographic profile of the WI and health predictors associated with PA participation a two-level multilevel regression model (individuals nested into their region) was fitted. This model investigated the interindividual variance in PA behaviour explained by variation in multiple deprivation in regions (random-slope), and baseline health outcomes and demographics (fixed predictors). Additionally, a series of multilevel longitudinal growth models [47] were used to examine the effectiveness of the programme. These models examined the interindividual variability (e.g., differences between members) within intraindividual patterns of change (e.g., time). The growth model nested time points (Level 1) into individual participants (Level 2). Both models were estimated through Iterative Generalised Least Squares and were constructed in sequential stages whereby a variance component only model was constructed to establish the interclass correlation coefficient (ICC). Following this fixed (model 1) and random (model 2) predictors were entered into the models. At each stage model fit was calculated through 2*loglikelihood and χ2 distribution tests for significance. General linear models were also constructed to investigate objectives relating to effectiveness and implementation. Specifically, data representing less than three time points (e.g., physical function) was analysed with a series of mixed-design (within-between) ANOVAs. Differences in demographics, PA and health profiles measured pre-baseline (e.g., those who signed-up for WN and do not sign up to the programme) were examined used independent samples t-tests (continuous variables) or χ2 distribution tests with Yates corrections for significance (categorical variables). Further, to understand aspects of reach and adoption descriptive statistics were utilised. A detailed overview of each the analysis process is available in Additional File 5.

### Findings

#### Reach

From March 2018 to January 2020 the WN programme reached (i.e., participated in at least one session) 3148 members (1.4% of the WI). Tailored advertising, trusted WI communication methods and face-to-face talks were effective in engaging participants. Advertising highlighted social support, common challenges to participation, accessibility of WN and adaptions to gameplay. Pre-baseline data indicates the mean age of WI members was 66.77 ± 7.22 years. Participants were married or in a partnership (80.6%), white British (99.4%) and 7.1% were in full-time employment. Almost half were degree educated (48.3%), and 49.5% had a household income less than £40,000. The mean multiple deprivation index score was 6.54 *±* 2.46. χ^2^ tests indicated a significant difference for partnership status (partner vs. no-partner; χ^2^ (1, *n* = 859) = 323.31, *p* = 0.001), ethnicity (White British vs. mixed ethic groups; χ^2^ (2, *n* = 859) = 1688.15, *p* = 0.001), employment status (employed vs. non-employed; χ^2^ (1, *n* = 859) = 222.32, *p* = 0.001), education (formal vs. no formal education; χ^2^ (5, *n* = 859) = 325.89, *p* = 0.001), and household income (χ^2^ (7, *n* = 859) = 276.52, *p* = 0.001). Demographic data is reported in Table 2 and bivariate correlations are presented in Table 3. WI members participated in an average of 3.55 *±* 2.13 days of PA per-week (i.e., > 30 min PA per day). The ICC of the variance only model was moderate (0.46), reflecting the need for a multilevel analysis [48]. The random-intercept model (model 1) explained 33.8% of the variance at the region level. A random-intercept and slope model (model 2) was constructed where PA at the regional level could vary as a function of multiple deprivation. Model 2 indicated 0.021 days behavioural variation was accountable to multiple deprivation across regions. This variability was not statistically meaningful (*p* = .15), and a χ^2^ indicated no meaningful difference between the models (χ^2^_*df = 16,*_ *p* = 1.0). Model fit statistics and parameter estimates are available in Table 4. All interaction effects between health outcomes and demographics were not statistically meaningful. Data recorded at pre-baseline indicates days per week of PA was significantly predicted by physical function (*β* = − 0.468, *p* = 0.008) and health status (*β* = − 0.557, *p* = 0.002).

**Table 2:** Pre-baseline participant demographic data

	Age Categories		
	Middle-Age (45–60 years) (***n*** = 155) (M ± SD, %)	Older-Age (> 60 years) (***n*** = 704) (M ± SD, %)	Total (n = 859) (M ± SD, %)
**Participant characteristic**			
Age	54.67 ± 3.63	69.43 ± 5.51	66.77 ± 7.22
Physical activity guidelines met	82.6%	81.3%	81.5%
Marital status (partner vs. no-partner)	83.9 vs. 16.1%	80% vs. 20%	80.6% vs. 16.1%
*Married or partnership*	76.8%	80.0%	80.6%
*Single*	7.1%	2.3%	3.2%
*Divorced*	6.5%	7.4%	7.2%
*Widowed*	2.6%	10.4%	9.0%
Employment Status (non vs. employed)	(23.2% vs. 76.8%)	(86.9% vs. 13.1%)	(24.6% vs. 75.4%)
*Full-time employment*	28.4%	2.4%	7.1%
*Retired*	9.0%	77.6%	65.1%
*Carer, volunteer, unemployed*	14.1%	9.4%	9.9%
*Part-time or self-employment*	48.4%	10.6%	17.5%
Ethnicity			
*White British*	99.4%	99.4%	99.4%
*Mixed ethic groups*	.6%	.6%	.6%
Education			
*Higher-education (*e.g.*, degree)*	55.5%	46.7%	48.3%
*Further-education (*e.g.*, A-Level)*	28.4%	25.9%	26.4%
*Primary-education (*e.g.*, GCSE)*	15.5%	21.3%	20.3%
*No formal education*	.6%	6.1%	5%
Social economic status (household income)			
*£ < 10,000*	1.3%	3.3%	2.9%
*£10,000–£20,000*	9.7%	15.8%	14.7%
*£20,000–£30,000*	12.9%	18.8%	17.7%
*£30,000–£40,000*	9.0%	15.6%	14.5%
*£40,000–£50,000*	9.7%	8.0%	8.3%
*£ > 50,000*	30.3%	11.1%	14.6%
*Unclear or not declared*	27.1%	27.6%	27.3%
Multiple deprivation index	5.91 ± 2.72	6.68 ± 2.38	6.54 ± 2.46

*Note*: M = mean. SD = standard deviation. % = percentage. Multiple deprivation index 1 = most deprived, 10 = least deprived

**Table 3:** Descriptive statistics and bivariate correlations between variables

Variable	M ± SD	***r(p)***											
		1	2	3	4	5	6	7	8	9	10	11	12
1. DpW PA	3.55 ± 2.13	–											
2. MHWB	52.80 ± 7.73	.12(.001)	–										
3. SI	3.01 ± 0.54	.11(.003)	.60(.001)	–									
4. QOL (physical fitness)	2.35 ± 0.93	−.29(.001)	−.16(.001)	−.11(.001)	–								
5. QOL (feelings)	2.14 ± .96	−.13(.051)	−57(.001)	−.53(.001)	.05(.12)	–							
6. QOL (daily activities)	1.59 ± 0.81	−.21(.001)	−.44(.001)	−.38(.001)	.32(.001)		–						
7. QOL (social activities)	1.44 ± .78	−.18(.001)	−.43(.001)	−.39(.001)	.23(.001)	.43(.001)	.71(.001)	–					
8. QOL (pain)	2.25 ± 1.0	−.13(.001)	−.19(.001)	−.14(.001)	.24(.001)	.45(.001)	.47(.001)	.47(.001)	–				
9. QOL (changes in health status)	2.87 ± 0.52	−.03(.032)	−.13(.001)	−.09(.007)	.07(.045)	.19(.001)	.08(.01)	.08(.001)	.10(.004)	–			
10. QOL (health status)	2.53 ± 0.87	−.31(.001)	−.41(.001)	−.31(.001)	.37(.001)	.09(.005)	.45(.001)	.45(.001)	.39(.001)	.42(.001)	–		
QOL (social support)	1.86 ± 1.09	−.07(.698)	−3.8(.001)	−.46(.001)	.07(.039)	.30(.001)	.23(.001)	.21(.001)	.11(.001)	.08(.019)	.21(.001)	–	
QOL (general)	2.13 ± .52	−.30(.001)	−.57(.001)	−.52(.001)	.50(.001)	.60(.001)	.77(.001)	.72(.001)	.63(.001)	.28(.001)	.69(.001)	.52(.001)	–

*Note:* M = mean. SD = standard deviation. Days per-week measured through single item of physical activity (30′ per day). QOL = quality of life. DpW PA = Days per-week physical activity. MHWB = Mental health and wellbeing. SI = Social Isolation

**Table 4:** Multilevel regression model predicting physical activity behaviour

Variable	***Model***	ICC	-2ll (^**df**^***p***-value)	***u***_**0*j***_	***u***_**2*j***_	***e***_**0*ij***_	***β***(SE)	***p***-value
	*Null Model*	.46	3649.48	2.04	2.37	–	3.42(.08)	–
	*Model 1*	–	3560.32 (^*df = 15*^ *p = .001*)	1.29	2.54	–	–	–
	*Model 2*	–	3558.84 (^*df = 15*^*p* = 1.00)	1.29	.021	2.42	–	–
Intercept (constant)							3.55(.169)	.001
Age							.004(.011)	.36
Multiple deprivation index							.017(.027)	.26
Mental health and wellbeing							−.016(.012)	.09
Social isolation							−.019(.165)	.45
*Quality of Life:* Physical Function							−.468(.195)	.008
*Quality of Life*: Feelings							−.187(.196)	.17
*Quality of Life:* Daily Activities							−.142(.213)	.25
*Quality of Life:* Social Activities							.207(.216)	.17
*Quality of Life:* Pain							.048(.191)	.40
*Quality of Life:* Changes in Health Status							−.018(.218)	.46
*Quality of Life:* Health Status							−.548(.198)	.002
*Quality of Life:* Social Support							−.061(.197)	.37
*Quality of Life:* Overall perceptions							.557(1.41)	.34
Employment Status *(reference: not employed* vs. *employed)*							−.093(.183)	.30
Partnership *(reference: alone* vs. *partner)*							−.148(.18)	.20

Note: ICC = interclass correlation. -2ll = −2*loglikelihood (deviance in IGLS estimation). Region level (*u*_0j_ = intercept, *u*_2*j* =_ slope). Member level (e_0*ij*_ = intercept

##### Who participates in walking netball?

Data measured at start of the programme indicates the average age of participants who sign-up to WN is 66.25 ± 8.74 years and 30.0% of WI members who sign-up to WN have a household income less than the median UK average (£30,800) [49]. Analysis indicates no statistical differences exist between members who sign-up and do not sign-up to WN in terms of age, ethnicity, marital status and educational history, and across markers of PA (metabolic equivalent of task; MET), mental health and wellbeing, and social isolation. However, meaningful differences do exist in markers of quality of life such as 6.0% lower physical function (t = − 2.32, *p* = .02), 6.6% lower perceptions of daily activities (t = 2.16, *p* = .03), 8.8% lower perceptions of social activities (t = 2.65, *p* = .008), 2.7% lower perceptions of health variability (t = 2.60, *p* = .009), and 7.4% lower perceptions of the ability to seek social support (t = 2.05, *p* = .04) in members who participated in WN. WI members may have sought participation in WN to improve health and quality of life and adapt to the aging process:

*‘I was very conscious of the fact I’m one of those people who had avoided sport all their lives and was well known, you know, for getting out of sport at school, never done any sport. As I hit middle life, I mean with menopause and things, I was starting to feel quite depressed, so, you know, the information is out there enough that sport does you good. So I started doing a bit of walking, realised actually it made me feel better, so when this walking netball came along, I thought, you know, how hard could that be? I should be able to do that without making a total idiot of myself, and that was a big fear’*

WI WN Member, Aged 73, Midlands

#### Effectiveness

##### Effectiveness on health outcomes

Data (see Table 5) indicates participation in WN has a meaningful positive impact on markers of mental health and wellbeing at 3-months (*b* = 1.15, *p* = .001), 6-months (*b* = .96, *p* = .001) and 12-month (*b* = .87, *p* = .001), loneliness at 12-months (*b* = −.045, *p* = .001), physical function at 3-months (*b* = −.10, *p* = .001) and 12-months (*b* = −.011, *p* = .001), feelings at 3-months (*b* = −.098, *p* = .001) and pain at 3-months (*b* = − 08, *p* = .001). A detailed overview of our findings representing the multiple-baseline study is presented in Additional File 6.

**Table 5:** The impact of Walking Netball on markers on psychosocial health, physical activity behaviour and quality of life

	ICC	Intercept (PB)	Baseline	3-months	6-months	12-months	Age	SES	Random Slope
*Mental health and wellbeing*									
Null model *b*(se)	.76	53.54(.42)*	–	–	–	–	–	–	–
Random intercept and slope *b*(se)	–	52.94(.49)*	.16(.28)	1.15(.28)*	.96(.37)*	.87(.44)*	−.05(.05)	.71(.32)*	2.31(.28)*
*Loneliness*									
Null model *b*(se)	.83	1.93(.02)*	–	–	–	–	–	–	–
Random intercept and slope *b*(se)	–	1.95*(.03)*	−.011(.014)	−.025(.017)	−.018(.02)	−.045(.02)*	.002(.003)	−.045(.021)	.008(.001)*
*QOL: physical function*									
Null model *b*(se)	.63	2.40(.04)*	–	–	–	–	–	–	–
Random intercept and slope *b*(se)	–	2.45(.05)*	.04(.03)	−.10(.04)*	−.08(.05)	−.011(.06)*	.00(.00)	−.10(.03)*	.04(.00)*
*QOL: feelings*									
Null model *b*(se)	.70	2.14(.05)*	–	–	–	–	–	–	–
Random intercept and slope *b*(se)	–	2.18(.06)*	−.03(.04)	−.098(.04)*	−.01(.05)	−.02(.06)	.00(.00)	−.11(.03)*	.04(.00)*
*QOL: daily activities*									
Null model *b*(se)	.61	1.53(.03)*	–	–	–	–	–	–	–
Random intercept and slope *b*(se)	–	1.55(.04)*	−.01(.06)	−.06(.03)	−.02(.04)	−.01(.05)	.00(.00)	−.05(.02)	.03(.00)*
*QOL: social activities*									
Null model *b*(se)	.64	1.39(.03)*	–	–	–	–	–	–	
Random intercept and slope *b*(se)	–	1.36(.04)*	−.00(.03)	.00(.03)	.09(.04)*	−.02(.02)	.00(.00)	−.02(.00)	.02(.00)*
*QOL: pain*									
Null model *b*(se)	.74	2.16(.05)*	–	–	–	–	–	–	–
Random intercept and slope *b*(se)	–	2.19(.06)*	.00(.03)	−.08(.04)*	−.08(.05)	.04(.06)	.00(.00)	−.10(.03)*	.04(.00)*
*QOL: changes in health status*									
Null model *b*(se)	.40	2.82(.02)*	–	–	–	–	–	–	–
Random intercept and slope *b*(se)	–	2.79(.04)*	.03(.03)	−.01(.03)	.03(.04)	.07(.05)	.00(.00)	.00(.00)	.03(.00)*
*QOL: health status*									
Null model *b*(se)	.75	2.45(.04)*	–	–	–	–	–	–	–
Random intercept and slope *b*(se)	–	2.49(.05)*	−.01(.03)	−.05(.03)	−.05(.03)	−.05(.04)	−.00(.00)	−.03(.03)	.02(.00)*
*QOL: social support*									
Null model *b*(se)	.73	1.70(.05)*	–	–	–	–	–	–	–
Random intercept and slope *b*(se)	–	1.78(.05)*	.00(.03)	−.04(.04)	−.04(.04)	−.00(.05)	.00(.00)	−.13(.03)*	.02(.00)*
*QOL: overall*									
Null model *b*(se)	.78	2.07(.02)*	–	–	–	–	–	–	–
Random intercept and slope *b*(se)	–	2.09(.03)*	.00(.01)	−.05(.02)*	−.01(.02)	−.00(.02)	.00(.00)	−.07(.02)*	.00(.00)*
*MET physical activity per-week*									
Null model *b*(se)	.61	2688.3(115.0)	–	–	–	–	–	–	–
Random intercept and slope *b*(se)	–	2547.0(147.8)	56.3(106.0)	188.3(118.0)*	29.9(135.8)	298.6(157.3)*	12.1(13.9)	−40.3(86.3)	226,934.13(35,275.5)*

*Note: Note:* PB (pre-baseline), *b* (regression coefficient), se (standard error), QOL (quality of life), ICC (Interclass correlation coefficient), * = *p* = <.05, MET (metabolic equivalent of task)

##### Effectiveness on PA behaviour

Participation in the programmes contributed to meaningful statistical improvements in PA MET-mins per week at 3-months (*b* = 188.3, *p* = .001) and 12-months (*b* = 298.6, *p* = .001). Likewise, mean minutes of PA per week improved from the start of programme (153.2 ± 110.7) to 3-months (*b* = 9.60, *p* = .044) and from 6-months to 12-month (*b* = 14.48, *p* = .032). At the start of the programme 47% of participants met PA guidelines [1] (i.e., > 150-min moderate-vigorous PA per week). The programme contributed to a 2% increase at 3-months, a 1% increase at 6-months and 6% increase at 12-months in the number of participants meeting these guidelines.

##### The effectiveness of the programme over medium-term on markers of physical function

**Fig. 1:**
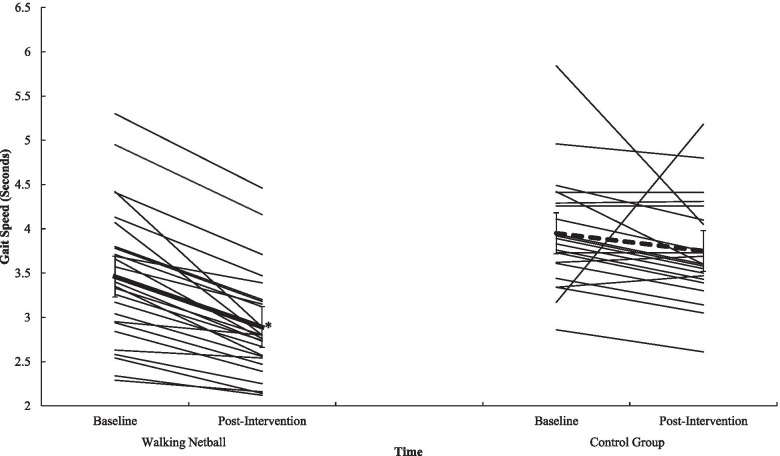
Interaction effect between T^0^ and T^1^ for Walking Netball and control groups on gait speed. Individual participants are represented with thin black lines. Group value represented with a dashed line. Error bars represent standard error. ^*^ = *p* = <.05

**Fig. 2:**
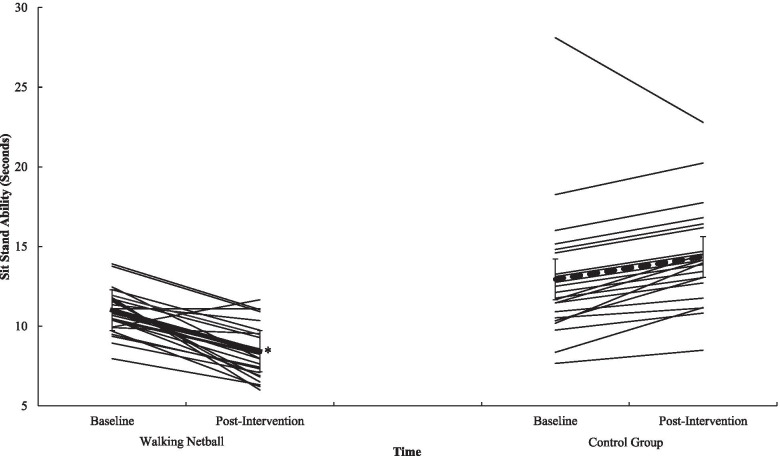
Interaction effect between T^0^ and T^1^ for Walking Netball and control groups on sit stand ability. Individual participants are represented with thin black lines. Group value represented with a dashed line. Error bars represent standard error. ^*^ = *p* = <.05

**Fig. 3:**
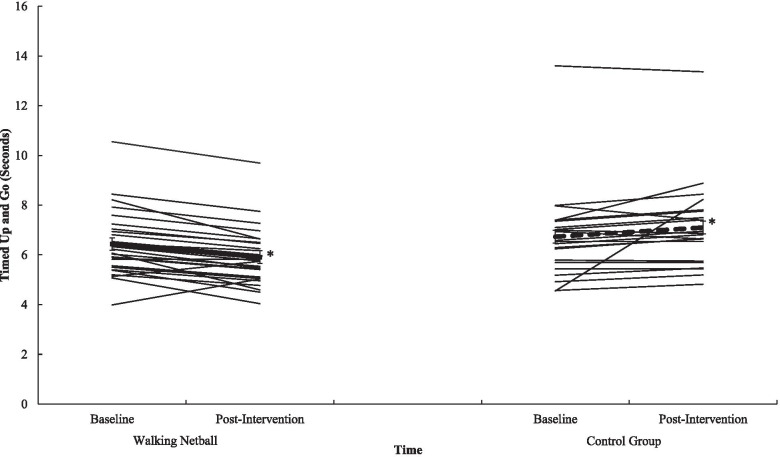
Interaction effect between T^0^ and T^1^ for Walking Netball and control groups on timed up and go. Individual participants are represented with thin black lines. Group value represented with a dashed line. Error bars represent standard error. ^*^ = *p* = <.05

**Fig. 4:**
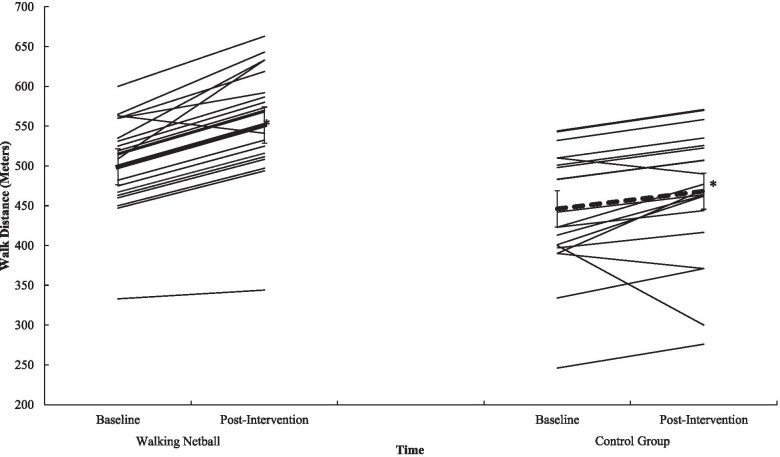
Interaction effect between T^0^ and T^1^ for Walking Netball and control groups on physical fitness (six-minute walk test). Individual participants are represented with thin black lines. Group value represented with a dashed line. Error bars represent standard error. ^*^ = *p* = <.05

Participation in WN contributed to statistically meaningful improvements in gait speed (*p* = .012, *n*^*2*^_*p*_ = .13) (see Fig. 1), in sit and stand ability (*p* = .001, *n*^*2*^_*p*_ = .59) (see Fig. 2) in balance scores (*p* = .009, *n*^*2*^_*p*_ = .04), in functional ability as measured through the TUG (*p* = .012, *n*^*2*^_*p*_ = .13) (see Fig. 3), and in physical fitness (measured with the 6MWT) (*p* = .001, *n*^*2*^_*p*_ = .63) (see Fig. 4) when compared to the control group. However, the programme did not contribute to meaningful adaptions in grip strength (*p* = .87, *n*^*2*^_*p*_ = .00). A detailed overview of findings (see Table 6) (including post-hoc differences) representing physical function is presented in Additional File 7.

**Table 6:** Physical function data for the Walking Netball and the control groups assessed at baseline (T^0^) and end of the intervention (T^1^)

	T^0^		T^1^		F Statistic(*n*^*2*^_*p*_*, p*)		
	M	SD	M	SD	Group x Time	Time	Group
*Gait-speed (Sec)*	3.69	.74	3.28	.72	6.87(n^2^_p_ = .130, *p* = .012)	29.98(n^2^_p_ = .395, *p* = .001)	14.56(n^2^_p_ = .240, *p* = .001)
Walking Netball	3.46	.76	2.89	.58			
Control	3.95	.64	3.75	.58			
*Sit-stand test (Sec)*	11.89	3.15	11.20	3.87	64.85(*n*^*2*^_*p*_ = .59, *p* = .001)	4.97(*n*^*2*^_*p*_ = .10, *p* = .003)	26.12(*n*^*2*^_*p*_ = .37, *p* = .001)
Walking Netball	11.00	1.43	8.43	1.68			
Control	12.94	4.20	14.35	3.18			
*Static balance test (0–4)*	3.63	.70	3.75	.60	4.50(*n*^*2*^_*p*_ = .009, *p* = .04)	2.27(*n*^*2*^_*p*_ = .05, *p* = .14)	3.23(*n*^*2*^_*p*_ = .07, *p* = .008)
Walking Netball	3.69	.55	3.96	.20			
Control	3.55	.86	3.50	.80			
*SPPB*	10.88	1.27	10.88	1.30	10.03(*n*^*2*^_*p*_ = .18, *p* = .001)	.07(*n*^*2*^_*p*_ = .001, *p* = .79)	8.26(*n*^*2*^_*p*_ = .15, *p* = .01)
Walking Netball	11.15	.83	11.46	.81			
Control	10.55	1.60	10.18	1.44			
*Timed up and go (Sec)*	6.57	1.58	6.45	1.58	20.65(*n*^*2*^_*p*_ = .31, *p* = .001)	.58(*n*^*2*^_*p*_ = .01, *p* = .45)	2.94(*n*^*2*^_*p*_ = .06, *p* = .09)
Walking Netball	6.43	1.35	5.91	1.18			
Control	6.73	1.84	7.10	1.77			
*Grip strength (Kg)*	22.97	6.13	24.06	5.84	.03(*n*^*2*^_*p*_ = .00, *p* = .87)	11.51(*n*^*2*^_*p*_ = .20, *p* = .001)	7.92(*n*^*2*^_*p*_ = .14, *p* = .001)
Walking Netball	24.94	6.23	26.08	5.83			
Control	20.55	5.17	21.58	4.91			
*6-min walk test (M)*	476.60	66.53	516.06	80.60	84.69(*n*^*2*^_*p*_ = .63, *p* = .001)	14.05(*n*^*2*^_*p*_ = .22, *p* = .001)	14.15(*n*^*2*^_*p*_ = .22, *p* = .001)
Walking Netball	498.93	51.07	551.19	61.20			
Control	446.15	73.91	468.16	80.25			

Note: Sec = Seconds. SPPB = Short Physical Performance Battery total score (higher scores reflect greater function). Kg = Kilograms. M = metres

##### Experiences and narratives representing effectiveness

Participation in the WN positively influences psychosocial health, quality of life, physical function, mental health, confidence and cognition. WN supported competence through providing a skill to master and achieve, reduced stress through distraction, happiness and enjoyment, and improved confidence. Adaptions in confidence were attributed to changes in functional movements, balance, mobility and functional motor skills. Maintenance of these markers during the aging process enables daily and social activities, reduces pain and variability in health. Further, elements of social processes such as friendship, teamwork and social connections along with identity and belonging were embodied during participation and consistently reported:

*‘It’s friendship, it’s fun. I think one or two ladies might be a bit stressed. I am very aware of that in our WI that you know a lot of ladies are bereaved, they’ve lost their husbands and then hence come to WI. It does alleviate that stress, worry and provides friendship and togetherness, you know, you feel a team I suppose is the word, teamwork’*

WI WN Member, Aged 72, North of England

#### Adoption

##### Setting up WN

WN was delivered within 46 (82.0%) of the 56 WI English regions. WN has been registered at 154 (2.4%) WI groups. Setting-up WN is dependent on the intrapersonal (e.g., age, ability, health), interpersonal (e.g., leadership, culture, support) and environmental (e.g., facilities, organisation) characteristics of each group. Additional costs, the ability of members, willingness to initiate a WN programme, insurance and facilities could hinder set-up. These challenges reduced the acceptability and feasibility of the programme for WI hosts. Where WN was successfully set-up, a flexible approach to funding and facilities was adopted. Further, outlining the expectations and outcomes of WN was important to the WI and its members. Supporting the set-up with Frequently Asked Questions (FAQ) and resources provided the WI with confidence and improved sustainability.

##### Working with the WI

Communication, stakeholder influence (i.e., the president, committee, hosts), and micro-politics within the WI shaped the social acceptability of WN. Indeed, no ‘perfect’ approach to communication was identified. Whilst some WIs were comfortable with digital communication, others preferred a formal and face-to-face approach. The latter challenged the logistics of WN hosts setting-up the programme across multiple regions. An approach incorporating email and a face-to-face group meeting was the most effective in encouraging sign-up. Central to WN was a proactive president, committee or WI member. A proactive host could influence participation. In contrast, the attitudes of a WI president who held a negative perception of sport, netball or PA could influence the behaviour of the members through removing the groups’ participation or proposing alternative activities to WN.

##### Where can WN take place?

The size, quality, availability and costs of a facility shaped WN participation. Inappropriate settings hindered participation and what happened in the early phases of the programme and the quality of delivery shaped the likelihood a WN would be maintained. Indeed, the cost of specialist spaces within regions and funding facilities over the long-term caused the programme to cease or be adapted:

*‘One of the barriers for me is venues, and which is why my sessions both folded, because they couldn’t afford the venues, and they worried then about having to pay out of the WI pot rather than the walking netball pot, to pay that venue, and that really worried them, and then that’s why they just pulled it. …… those are the types of sessions that struggle a bit more when a sports hall is asking me during the day for nearly £60 for an hour, and that’s just not… it is not within the nature of what we’re trying to achieve within the project. And I think that’s another massive, massive barrier, the finances of it’*

WN Host, Aged 36, South of England

##### Identifying a host

A proactive host who was committed to delivering WN, was outgoing and extroverted, and could lead and communicate in sessions was perceived to be the most effective in their role. Remaining proactive improved the effectiveness of the implementation process:

*‘I’m quite lucky that they’re a very proactive host and on the ball themselves, which is quite helpful. They’ve got two people that have already done their host training for preparation for when they take it over, so yeah, no it’s going well, yeah. They’re quite proactive, there’s two of them that are working quite closely together and then they have the treasurer of the WI, so all of the … she comes along, she’s a hoot this woman, she’s just nuts, she’s brilliant, love her! But she sort of comes along, she does their registration, collects all the money’*

WN Host, Aged 29, Midlands

In contrast, some WN hosts highlighted challenges such as confidence and logistics when identifying WI hosts. To address these challenges WN hosts adopted an approach where multiple WI hosts were trained to lead WN. Multiple hosts provided consistency yet allowed each host to participate.

##### Training the WI to host WN

Host training was delivered face-to-face by England Netball in central regional locations. This training day was delivered alongside netball coaches, umpires and development officers. Training covered the rules of WN, health and safety, skills and drills, adapting gameplay, umpiring, leading sessions, and remaining confident. To date, 228 hosts have been trained. Accessible training (e.g., local, funded, logistically viable), alongside a willingness to provide a supportive experience were enablers to attending host training. However, teaching WI hosts alongside knowledgeable and experienced netball participants challenged perceptions of confidence. With this said, WI hosts shared the same motivation to help others and their experience was deemed valuable by others. Host training was both theoretical and practical. Practical tasks alongside other hosts further improved delivery:

*‘They did a very good exercise where this woman just grunted at you and didn’t really speak to you and things like that, which made you feel like ‘well I don’t want to bother,’ and they were like “So that’s what you don’t want to do.” So that was quite good. We learnt lots of the warmup games. We were all given a turn to take one of them so that you learn actually you need to give clear instruction because if you don’t people won’t understand it and you need to make sure that everybody understands it’*

WI Host, Aged 64, East of England

Practical tasks provided translatable exercises WI hosts could implement into their WI. Host training was effective at influencing delivery. Hosts reported confidence when leading components of the session. The training allowed WI hosts to engage WI members, prevent injury, and adapt drills and games (e.g., for differences in age, health status and ability).

##### WI adoption

Of the 154 WN groups set up, 135 (87.7%) WN groups’ were maintained beyond the 20-session initial delivery phase. WN sessions varied between the WIs. Warm up activities involving walking and catching were designed to elicit physiological adaptations, promote functional motor skills and create an engaging social activity where new members could be introduced, and relatedness could be supported. Skills and drills focused on core skills such as shooting, passing, movement and game play in a progressive and inclusive style. Tailoring to specific issues such as reduced motor control, reinforcing correct technique and preventing injury were widely reported. Games rarely followed a competitive structure. Rather, members switched teams, rules were adapted, and time limits were not observed. Adaptions encouraged relatedness and improved competence:

*‘We couldn’t play in the, you know, the full game, the running game, people are perhaps recovering from illness, operations or whatever, or even, you know, just having... having age on their side and not being able to do as much and haven’t got as much mobility. And I just thought WN just lends itself to anybody and everybody with or without ability. You can just get out there and just do it. You know, there was no pressure. We amended the rules to help. You know, basically you walk, walk everywhere and then, you know, you get an extra step, an extra second to hold the ball. It’s all geared for slowing down the whole game and it’s great. It’s just great to be able to do it’*

WI Host, Aged 60, North of England

#### Implementation

##### What determines participation?

**Fig. 5:**
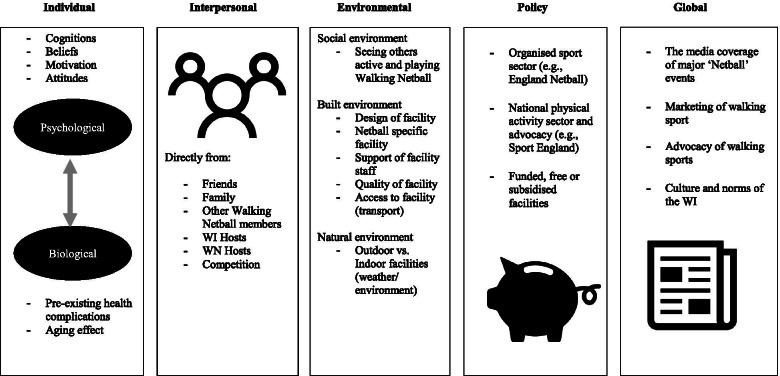
The Walking Netball Social-Ecological Model. *Note:* Social-ecological model of physical activity (Bauman et al., 2011). Adapted for Walking Netball

Participation in the WN programme is determined by factors present across a social-ecological model [50] (see Fig. 5). Global-level factors such as an awareness of ‘walking’ sports, netball knowledge, mass sport events (e.g., Commonwealth Games, Netball Super League, World Cup), and the purpose and culture of the WI shaped participation. Within the UK, netball is a historic component of the school-curriculum. Historic experience led to a basic understanding and awareness of the rules of Netball. Netball knowledge held the capacity to facilitate self-efficacy and perceptions of competence. In addition to success on a global stage England Netball have popularised a domestic ‘Super League’. These events served to raise awareness surrounding participation. Members reported motivation via identity and belonging. Further, walking sports have grown in popularity within the UK. More specifically, participation in walking sports such as soccer have been promoted nationally by charities, governing bodies and mass-media organisations. Participants reported how continued ‘sport for all’ promotions normalised their participation in WN. Support and awareness on the global-level were further fostered by the values of the WI (i.e., providing opportunity, growth, education and development to women). These aims are embodied by the culture of the WI and by those who organise events, campaigns and promotions. The WI’s culture provides encouragement and investment for members who engage in WN. Further, WI members and hosts commented on their awareness of the programme through the support of the central WI and its regional federations. Organisational support and investment were reported to be associated with feelings of integration and belonging and a source of maintained participation.

The setting in which WN was conducted and the policies associated with these facilities modified participation. A funded or subsidised facility could encourage participation, while an expensive or over-subscribed facility could challenge participation. WI groups who negotiated with Active Partnerships (i.e., a national network of organisations who work with local partners to promote sport and PA), leisure centre providers or councils to subsidise or fund the use of a facility consistently reported high attendance. Further, WI groups worked with local schools or played on a free to use outdoor court. Access to these facilities promoted participation during warmer months, however challenged engagement during winter. Access and the quality of a facility could shape participation. For example, early phase WN was delivered in church or village halls or WI buildings. Whilst accessible, these buildings often challenged participation and hosting due to a lack of netball court markings, netball goals or non-slip floors. These challenges could create confusion, reduce competence and exacerbate injury. To some extent avoiding using village or church halls, a WIs building or small spaces could be averted by participation outside or managed through resources provided by England Netball (e.g., non-slip floors, equipment). Consistently, members playing within an appropriate facility reported competence and high self-efficacy.

Competence and self-efficacy were both reported to be enabled by direct and indirect social support. Indirect social support is on the environmental-level and is vicarious. WN and WI hosts found participation in WN could be supported using a progressive observational style where members were integrated into direct elements of the programme over time. This style of delivery could be further reinforced with videos prior to participation. Directly, social support was provided by hosts, family, friends and WI. Hosts supported the psychological basic needs of autonomy, competence and relatedness throughout WN [51, 52]. This included tailoring sessions to individual members, offering choice and control to members and creating a socially vibrant setting where enjoyment was valued over competition. Importantly, hosts were approachable and enthusiastic about WN:

*‘I think she’s just a natural people person. She’s really warm, friendly. She’s got a twinkle in her eye. You know, we have quite a lot of laughs with her. She’s very fun. She like blows the whistle for bad behaviour! It’s like having that sense of fun, isn’t it, really? And she just keeps it flowing as well. The whole thing’s not hard work, it just flows. Like the hour just goes really quickly’*

WI WN Member, Aged 74, Midlands

Directly, family and friends supported participation through reinforcing competence, providing transport to sessions or acting as role models. WI members appeared to support participation through social engagement and friendship or challenge participation through competition. Consistent with school-sport, some members were motivated by the external achievements and demonstration of competence associated with competition [53], while others felt dejected, and their competence was challenged through its physicality, intensity and meaning:

*‘At one point I almost stopped because I did feel it was getting too competitive. I mean like the other day when it got too fast, didn’t it? The ball was flying about all over the place and Julie said “Stop! Before you pass it on you’ve got to bounce it.” So she found a way of slowing us down because it was getting too fast and, you know, that just made us realise “Oh, hang on a minute, it is supposed to be walking and we have got a range of abilities, haven’t we?” So we have to make sure we cater for the whole range of abilities, not just the ones that are good or the ones that are quick or you know. It’s got to be enjoyable for everybody’*

WI WN member, Aged 76, South of England

On an individual-level injuries and their impact could challenge participation in the short- and long-term. Some members dropped out of WN due to identifying participation with the potential for injury. Injury in aging adults is a concern, it prevents long-term movement and reduces quality of life. WI members expressing these concerns discussed balancing the risk and reward of participation. This perception to an extent was altered through vicarious experiences of WN and support of hosts. Minor injuries (e.g., fractured fingers, hands wrist, collarbone, muscular strains and sprains) were often the result of differing abilities and styles of play:

*‘Some of the ladies would only walk and they were a lot slower than I was and they’d all been told about me and how sort of…not fierce I was, but how competitive I was. When I watched some of the ladies I thought “Why can’t I be like them and just take it at a much slower pace?” But that’s not in my nature, unfortunately. And I think we’re all different and however much you try and say “Walk, walk, walk,” it still doesn’t slow you down because your mind is playing games with you and you want to get that ball because you don’t want to let the team down’*

WI WN Member, Aged 61, Midlands

Injury, when it occurred, could reduce participation for both the affected member and their WI group. Members reported dropping out following their peer being injured. The way in which a host managed an injury, both during the early and later stages, determined future participation. For example, remaining professional and adapting practice to prevent further injury enabled participation. Adapting participation included a progressive warmup, prompting walking, and correct patterns of movement.

Hosts attempted to support autonomy through providing choice and control [51, 52]. Competence could be supported through adapting drills, breaking down games, removing and adapting rules, simplifying complications, using crib cards and resources, and reinforcing important rules such as walking and competition [51, 52]. Hosts and the environment in which WN was conducted could support relatedness. Social support, conversation and regular connections could be enhanced through both participation in WN and the surrounding context (e.g., coffee, driving to the venue, breaks between play) [51, 52].

Self-efficacy shaped by a behavioural, cognitive and environmental sources influenced WN participation. Fitness, health status and physical function could determine self-efficacy to participate in the programme. Health status and current PA participation encouraged self-efficacy and likewise participation in WN. In contrast, reduced perceptions of physical function, health status and fitness could reduce self-efficacy and subsequently participation in the programme. These challenges to self-efficacy could be overcome through social support:

*‘If you haven’t done sport for a long, long time you think “Oh, I can’t do that,” you know, but when you actually get into it and get the enthusiasm and the encouragement from everybody else……. And I’ve got a lot of physical problems. To be actually able to go and do that and feel part of it, it’s brilliant’*

WI WN Member, Aged 80, East of England

##### Participation and satisfaction of WN

Session attendance across 2556 members varied widely (11 ± 13 sessions completed). Satisfaction ranged from 1 (not satisfied) to 5 (completely satisfied). Findings indicate participants were completely satisfied with the programme at 3-months (59.0%), 6-months (56.0%) and 12-months (52.0%). Average satisfaction declines marginally over time (F_(1.67, 5.11)_ = 5.895, *p* = .005, n^2^_p_ = .019). Follow up testing suggests satisfaction with the WN programme declines significantly between 3-months and 6-months (−.186, *p* = .016), and 3-months and 12-months (−.253, *p* = .016). However, it should also be noted satisfaction with the programme maintained a high mean average (4 ± 1.4).

#### Maintenance

Promotion within the local community, sustainable funding, inter-WI competitions, festivals and networks, multiple-hosts and continued host development can contribute to the maintenance of the programme. Promotion of WN beyond the WI held benefits not only for the maintenance of the programme but the WI itself. Broadening the reach of WN with local communities provided a sustainable model for regular participation and reduced costs to hire facilities. Allowing WIs to advertise the programme within the community provided ownership, and a mission to offer opportunities to women within their local area. The latter is a core foundation of the WI’s purpose. Widening participation changed some perceptions of the WI, where membership had been perceived as exclusively for older-adults. A WI offering WN altered these perceptions, with women attending WN often going on to joining the WI. To sustain the programme WIs built relationships with Active Partnerships, leisure providers and created surplus funding. Relationships and surplus funding through charging members an increased cost per-session allowed WIs to cover costs, purchase equipment, fund trips and support members living below median national household income.

Alongside fiscal sustainability, the maintenance of the WN was improved through inter-WI competitions, WN festivals and virtual and face-to-face member networks. Sport is inherently associated with competition. Whilst competition could be a challenge for participation, in other cases competition was the next step in creating sustainable participation. Centrally England Netball organised WN festivals where WIs competed against each other. Further, proactive hosts organised local inter-WI games which incorporated social activities and broadened networks within the WI. WI networks or virtual networks for members and hosts (via Facebook) further reinforces social support, identity and belonging and creates a setting to share best practice (e.g., coaching drills, games, skills).

The adaption of WN to a range of experiences, backgrounds, health complications and levels of ability was a challenge for all WI hosts. Continued development pathways were therefore identified as important in maintaining the WN programme. WI hosts engaged in self-directed development through accessing online and written resources, watching YouTube videos, using social media and through host networks. Sharing drills and skills and adaptions was reported to improve the quality of sessions. To enhance netball knowledge, online resources, DVDs, booklets and courses were suggested alongside face-to- face training. A range of educational opportunities similar to coaching development pathways may promote a more inclusive training experience. With this said, it is important to note the WI hosts who delivered the programme were volunteers, with commitments outside of WN. An approach with multiple WI hosts (beyond the two trained through the programme) helped improve the quality of hosting, and reduced stress and pressure placed upon hosts. In addition, multiple WI hosts within a session allowed hosts to participate in WN rather than simply leading:

*‘It’s great because you can play, you don’t have to referee all the time. You can actually play yourself, so we take it in turns. There are just more people involved. Four of us are really keen and that just comes out I think to everybody else. It isn’t just one person trying to motivate everyone, it’s all of us. I’ve got grandchildren and sometimes there are other calls on my time, and I’ve got family who don’t live near me. And also obviously when you’re retired you don’t just do things in school holidays. Having four of us, there’s always someone to lead. I would suggest that anybody who’s trying to do it on their own really needs some back up because you never know what’s going to happen to you and you don’t want it to stop just because you can’t, you know, and then you’re running round trying to find someone to take your group and things like that’*

WI WN Host and Member, Aged 69, Midlands

WI members are at a greater risk of COVID-19. Therefore, adapting the programme both in terms of restrictions and for the recovery from COVID-19 was vital to the long-term maintenance of WN. Adapting the programme to the health, physical function and self-efficacy of members, re-training and supporting WI hosts, tailoring the re-start of the programme to individual groups and providing accessible resources are vital in the return of the programme. In the short- to medium-term the programme proved to be maintainable through virtual delivery via ‘Zoom’. From April 2020 a twice weekly session and stand-alone ‘Biggest Wiggle and Giggle’ and Christmas special of virtual WN was delivered. Alongside live delivery, sessions were uploaded to YouTube to improve the reach. Sessions included content similar to face-to-face WN, but adaptions for the home. The purpose of sessions was to maintain health and wellbeing, and netball knowledge. Streaming the sessions online encouraged social engagement through comments boxes on social media channels. To date, 7200 individuals have viewed virtual WN sessions. Virtual WN has led to new expressions of interest from WI groups not previously playing face-to-face WN. In addition, members reporting using a wall and ball to practice passing, working on footwork and balance, retrofitted goals and playing with family and members of a support bubble. This sense of identity and enjoyment highlights the long-term impact of WN on the behaviour and lifestyle of WI members.

## Discussion

The current study evaluated the acceptability, feasibility and efficacy of the WN programme via the RE-AIM framework [33, 34]. There is limited evidence representing the acceptability (i.e., reach) and feasibility (i.e., adoption or implementation) of sports-based programmes for middle- to older-adults. Previous community-based PA interventions have reported reach values (2.0–90.0%) higher than WN [53–57] and influence a more diverse population of middle- to older-adults [58]. WN and the WI has the potential to reach a greater heterogeneity of society such as those at risk of health inequalities. However, in the case of WN the advertising of the programme was tailored to promote participation to members currently situated within the WI and therefore can be considered socially acceptable. Promoting good treatment acceptability, WN reaches inactive members who report poor quality of life. Indeed, while mental health and wellbeing [59] and social isolation were higher than population norms [60], findings indicate WI members do not participate in sufficient PA to meet national guidelines designed to protect from ill-health [1] and report perceptions of quality of life lower than the general population [61]. Interestingly, whilst not meaningfully different in demographic markers and PA behaviour, members who sign up to WN report lower perceptions of quality of life than their peers who do not sign up to the programme, suggesting the programme has high treatment acceptability (i.e., participants perceive the programme will promote health). To some extent this level of acceptability may be attributed to the advertisement of WN. To facilitate treatment acceptability, advertising focused on common health complications, barriers to sport and the provision of social-support would be beneficial. These forms of advertisement may support self-efficacy [62], competence and promote relatedness [51]. Given previous research has often engaged those already active and healthy in sports and exercise based interventions [63], this finding should be highlighted as the strength of the programme.

WN was effective at encouraging uptake and maintenance across regions and groups in England. The implementation of WN was influenced by factors on the global-, policy-, environmental- and interpersonal-level such as awareness, set-up, host training and WI host delivery, communicating with the WI, facilities, identifying a WI host and social support. These factors manifested as determinants for participation on the individual-level as self-endorsed motivation and self-efficacy for participation. Whilst previous research has not investigated the process underpinning the context in which interventions are conducted, participation appear to be shaped in a similar extent by health status, the aging process, social community, competence, motivation, demographics, competition and programme organisation [13, 14]. Therefore, interventions designed to promote health in an aging population via sports-participation could improve acceptability and feasibility through practises adopted during the WN programme such as offering flexibility in setting-up, delivering and maintaining participation [64]. In the WN programme, adopting a needs-supportive approach to warm-ups, skills and drills and gameplay, tailoring practises to promote functional movement and injury reduction, and adapting venues and settings where WN was delivered proved to be successful. It should be noted that this degree of promotion and delivery requires meaningful organisation (e.g., tailored set-up), training of a WI host, and mentoring and resources (e.g., FAQs documents). Most importantly, when promoting community-based programmes, an appropriate champion (i.e., a WI host) must be identified and appropriately supported and trained. Data indicates this support is most feasible and effective through a blended face-to-face and virtual style.

Participation in WN contributed to improvements in mental health and wellbeing and reductions in social isolation over the long-term. The effectiveness of WN is comparable with studies using mixed-gender team sports (i.e., hockey and novel sports) which have reported 4.0% improvements in psychological wellbeing, 2.7% reductions in anxiety, 10.6% reductions in depression and no meaningful changes in social health outcomes over the short-term [18]. Findings are consistent with qualitative research exploring sports participation in an aging population, suggesting sports participation can create identity and belonging [25], promote subjective wellbeing [27], and facilitate healthy aging [23, 26]. A strength of community groups such as the WI are their ability to promote relatedness and social-support through membership [65]. WN offers a stable consistent setting to promote social belonging and support over the long-term [65]. Improvements in mental health and wellbeing as a function of a change in PA participation can occur due to biological, psychosocial, cognitive, psychological and neurological adaptions [66]. In the case of middle- to older-age women sports participation can promote an enjoyable experience, self-efficacy, competence, and develop identity and shape coping mechanisms with the aging process [14]. WN offers middle- to older-age women a set of skills to master, cognitive functioning, variable degrees of competition, social support and consistent pattern of activity, factors likewise associated with good mental health and wellbeing [66].

The programme improved objective physical function, and markers of quality of life. More specifically, participation in WN contributed to meaningful improvements in gait speed, balance, functional ability and physical fitness, but not muscular strength or overall function. Participation in WN contributes to a reduced risk to perceptions of physical function, and minor reductions to risk to feelings, pain and overall quality of life over the short-term, and risk to social activities over the medium-term. Previous research using objective measures has found sit-stand function and arm curl repetitions to improve following weekly participation in walking soccer over 12-weeks in untrained men and women [18, 19] and reduced body fat mass and percentage and improved increased time to volitional exhaustion during incremental treadmill testing [20]. Further, participation in mixed team sports (i.e., soccer, handball, volleyball, basketball) with male and mixed gender groups have been found to improve sit-stand ability and balance [21]. In contrast, sport with reduced freestanding movements such as floorball may not improve functional ability over the long-term [17]. In terms of quality of life, previous research has found similar size improvements in general (2.3%) and health related (5.5%) quality of life, but no meaningful changes in social and emotional aspects [18]. Our findings are consistent with research outside a sports context. More specifically, Tai Chi conducted over the short- to medium-term can result in small positive effects in quality of life markers relating to physical and emotional health outcomes in older-adults [67]; whilst participation in yoga has the capacity to improve markers of quality of life associated with mental health, physical function and sleep quality [68]. Data drawn from a systematic review of randomised control trials across a range of modes of PA indicates activities which were played at a moderate intensity, require high levels of cognition and involve social engagement offer the greatest benefits for quality of life [69]. Given WN is played at a moderate intensity, requires cognitive function and is centred around social engagement, along with our positive findings, it may be recommended as a mode of PA to promote good quality of life. Fostering quality of life through sport has the capacity to promote healthy aging [13].

WN participation contributed to a meaningful change in MET minutes of PA per week over the short- and long-term. A positive change in PA behaviour is associated with markers of mental health, quality of life and physical function. Meeting PA guidelines [1] and maintaining participation is an important indicator for health promotion programmes such as WN. Our data at pre-baseline indicates WI members did not participate in levels of PA recommended to protect from maladaptive changes to health and wellbeing. Maintained MET duration at recommended levels (i.e., 3000 MET-minutes per week) can contribute to reduced NCD incidence and all-cause mortality [11, 70]. Our findings suggest participation in the programme improves MET-minutes per week by 11.7%. Improving MET-minutes can further reduce the risk of NCD in middle- to older-age adults [11, 70]. Further, our findings indicate WN provides an accessible and feasible mode of PA to meet national guidelines for working-age (middle) and older-adults to reduce the risk of NCDs. WN offers a consistent mode of moderate- to vigorous-intensity PA across a social-ecology of behavioural determinants of participation and maintenance.

Whilst WI members were satisfied with the programme, the maintenance of WN is dependent on its long-term promotion and subsequent adaptations to the COVID-19 pandemic. Findings demonstrate that virtual forms of WN, whilst seemingly less effective than face-to-face delivery are acceptable and feasible in the short-term to medium-term. Future research may seek to understand the extent to which participation in virtual forms of PA delivery can maintain health and wellbeing outcomes, and motivation and self-efficacy for participation. Returning to WN whilst adapting to the impact of the pandemic is also important. To encourage WN and adapt to the changes associated with COVID-19, WIs may consider methods to promote sustainability such as promotion within the local communities and encouraging inter-WI networks. This process could be supported through partnership with local- and regional-level Active Partnership organisations. Support through these partners may provide further encouragement to hosts leading WN. Additionally, WI hosts may be further supported through recruiting additional hosts within the WI. In a similar style to peer led programmes within a workplace context, additional hosts may encourage participation and create sustainability [71]. Centrally, support to WI hosts may include additional workshops in similar style to coach education pathways. This training could be tailored to factors influencing the acceptability of promotion within middle- to older-age adults such as health status, healthy aging, and encouraging self-efficacy, motivation and social support.

### Strengths and limitations of the evaluation

The current study presents the first attempt to evaluate WN using the RE-AIM evaluation framework. Our use of this framework and robust mixed-methods approach is a strength of the study. The current study adds to a growing body of literature which explores the provision of walking sports for an aging population and addresses limitations regarding understanding the process of setting-up and delivering sport for middle- to older-age women. However, our study does have limitations. Indeed, the adoption of a randomised cluster control trial, whereby regions could be randomised into either an intervention or control may provide stronger evidence in terms of effectiveness. This design may include further measures beyond 18-months. In the case of the current study this was preferable but avoided for pragmatic reasons relating to our stakeholders. Further, the COVID-19 pandemic limited our measurement of some individuals over time. The pandemic resulted in WN sessions being suspended and therefore the collection of data regarding the programme. Moreover, whilst strong in the assessment of clinical predictors of physical function, the lack of randomisation, predictive measure of cardiorespiratory function, our lack of evaluation of the physiological bases for improvement, self-report assessment of covariates, and absence of a true control group and measures over the short- and long-term are a limitation. Finally, our sample only represents a small proportion of the WI members present within the England WI (Wales was not a funded element of the programme and Scotland and Northern Ireland are separate WIs).

## Conclusion

Participation in sports-based programmes may promote health and wellbeing in middle- to older-age adults [14, 18, 19]. The present study evaluated the acceptability, feasibility and effectiveness of WN to middle- to older-age women using the RE-AIM framework. A success of the programme was promotion to the target populations’ needs, barriers to participation and identity. This step proved effective at reaching members at risk of ill-health and is recommended for future programmes. The programme was feasible over the long-term due to adaptions made during set-up and delivery. Indeed, in a similar extent to WN, future programmes should adapt to people, places and spaces through needs-support and providing a range of resources. The acceptability and feasibility of the programme shaped its effectiveness on PA behaviour and health markers. The maintenance of PA behaviour may have contributed to positive adaptions in mental health, physical function and quality of life outcomes. Future research may seek to understand how participation in community-based sports programmes can maintain behaviour and health over the long-term and contribute to healthy aging. In summary, findings suggest walking sport and specifically WN may be an acceptable, feasible and effective method to promote health and contribute to a healthy aging process.

## Supplementary Information

**Additional file 1.** Measures recorded during the multiple-baseline study.

**Additional file 2.** Schematic overview of the quasi-experimental study.

**Additional file 3.** Physical function measures record during the quasi-experimental study.

**Additional file 4.** Qualitative analysis.

**Additional file 5.** Quantitative analysis.

**Additional file 6.** Findings: Multiple-baseline study.

**Additional file 7.** Findings: Quasi-experimental study - Does participation in Walking Netball (WN) improve extremity function and gait Function.

## Data Availability

Statistical data representing reach, adoption and efficacy is publicly available. Qualitative data representing each RE-AIM dimension is available on request. The datasets generated and/or analysed during the current study are available in the Open Science Framework repository, (https://osf.io/r4yqd/).
