# Supplementary material for: Is walking netball an effective, acceptable and feasible method to increase physical activity and improve health in middle- to older age women?: A RE-AIM evaluation
Source: Int J Behav Nutr Phys Act. 2021 Oct 19;18:136. doi: 10.1186/s12966-021-01204-w (PMC8524399; doi:10.1186/s12966-021-01204-w)
Supplement: Supplementary file 1 — Additional file 1. Measures recorded during the multiple-baseline study. [file 12966_2021_1204_MOESM1_ESM.docx]

**Additional File 1**

**Measures recorded during the multiple-baseline study**

***Mental health and wellbeing***

The Warwick-Edinburgh Mental Wellbeing Scale^1^ was used to examine mental health and wellbeing. The scale requests participants to respond to 14 Likert scale items ranging from 1 = none of the time, 5 = all of the time^1^. A total score is calculated ranging from 14 to 70, where higher scores reflect greater perceptions of mental health and wellbeing^1^. The measure has previously demonstrated a strong Cronbach’s Alpha score (α=.91)^1^.

***Loneliness and social isolation***

Perceptions of loneliness and social isolation were observed using the UCLA loneliness scale (version 3)^2^. Constructed from eight Likert scale item (1 = never, 4 = often) (two positive items reserved scored), the measure requests participants to rate their perception of loneliness^2^. A total sum score was first calculated. To improve translation to stakeholders this was re-calculated as a mean average score for all items. The measure reports strong internal consistency (α=.89-.94) and good re-test reliability (r=.73)^2^.

***Quality of life***

The Dartmouth COOP Functional Assessment Chart^3^ was used to examine quality of life across markers of physical function, feelings, daily activity, social activity, pain, health status, changes in health, social support and overall perceptions across a four-week period. Factors are weighted negatively on a 5-point Likert scale (1= not at all, 5 = extremely) where lower scores reflect reduced risk to quality of life^3^. The measure has reported good convergent and discriminant validity in previous research^3^.

***Physical activity behaviour***

Physical activity was measured using the International Physical Activity Questionnaire (IPAQ)^4^. The IPAQ is constructed from seven self-reported items which examine frequency and duration across vigorous- and moderate-intensity PA; walking and sitting^4^. Following a standardised scoring and truncation procedure^4^, duration and frequency were calculated as metabolic equivalent of task (MET)-minutes per-week^4^. The IPAQ has been found to provide valid predictions of objectively measured physical activity in older-adults^5^ and has demonstrated an acceptable Cronbach Alpha (α=.60)^6^.

**References**

1. Tennant R, Hiller L, Fishwick R, Platt S, Joseph S, Weich S, et al. The Warwick-Edinburgh Mental Wellbeing Scale (WEMWBS): Development and UK validation. Health Qual Life Outcomes, 2007;5; doi: 10/1186/1477-7525-5-6.
2. Russell DW. UCLA Loneliness Scale (Version 3): Reliability, validity, and factor structure. J Pers Assess. 1996;66:20-40; doi: 10.1207/s15327752jpa6601_2.
3. Nelson E, Wasson J, Kirk J, Keller A, Clark D, Dietrich A, et al. Assessment of function in routine clinical practise: Description of the COOP Chart method and preliminary findings. J Chro Dis. 1987;40:55S-63S; doi: 10.1016/S0021-9681(87)80033-4.
4. Craig CL, Marshall AL, Sjöström M, Bauman AM, Booth ML, Ainsworth BE, et al. International Physical Activity Questionnaire: 12 country reliability and validity. Med Sci Sport Exer. 2003;195:3508-1381. doi: 10.1249/01.MSS.0000078924.61453.FB.
5. Kolbe-Alexander TL, Lambert EV, Harkins JB, Ekelund U. Comparison of two methods of measuring physical activity in South African older adults. Journal of aging and physical activity. 2006;14:98-114.
6. Mannocci A, Di Thiene D, Del Cimmuto A, Masala D, Boccia A, De Vito E. International Physical Activity Questionnaire: validation and assessment in an Italian sample. Ital J Publ Health. 2012;7.
