# Supplementary material for: Is walking netball an effective, acceptable and feasible method to increase physical activity and improve health in middle- to older age women?: A RE-AIM evaluation
Source: Int J Behav Nutr Phys Act. 2021 Oct 19;18:136. doi: 10.1186/s12966-021-01204-w (PMC8524399; doi:10.1186/s12966-021-01204-w)
Supplement: Supplementary file 2 — Additional file 2. Schematic overview of the quasi-experimental study. [file 12966_2021_1204_MOESM2_ESM.docx]

**Additional File 2: Schematic overview of the quasi-experimental study**

**Pre-intervention tasks**

Women’s Institutes (WIs) recruited; assigned Walking Netball or control (waiting list); expression of interest from participants within WI groups. >40 participants required for meaningful effect (n=30 per group) (*d* =.50, 1-β =.95, α =.05, 2*2 ANOVA, >25% attrition rate)

**Enrolment**

**Inclusion/exclusion criteria applied**

**Included:** Over 45 years of age; able to participate in Walking Netball or attend WI meetings for 6-months.

**Excluded:** Not meeting inclusion criteria; pre-menopause; unable to provide informed consent.

**Eligibility:** Walking Netball group site (*n*=30) Control group (*n*=30)**.** Both groups of participants selected on first come basis.

**Screening/clustering**

**Measures**

**(T^0^) Baseline measures**

The Short Performance Battery (extremity and gait function)

The ‘Timed Up and Go’ test (extremity and gait function)

Grip strength and physical fitness (six-minute walk test)

**Intervention/control conditions**

**Control group**

- Continued with normal lifestyle
- Group physically active
- No participation in walking netball (6-month waiting list)
- Walking netball assigned post intervention
- Dropouts at post-measures

**Walking Netball Group**

- 20-weeks coach led netball
- 6-weeks host and coach led netball
- Sessions involve progressive warm-up, skills, drills, mini-games, and a full game
- Autonomy provided for breaks and progression
- Sessions last 60-90 minutes
- No dropouts

**Measures/analysis**

**Dropouts at T^1^**

Intervention (*n*=0), Control (*n*=8) (lifestyle demands, uncontactable)

**(T^1^) Outcomes measured and analysed**

The Short Performance Battery (extremity and gait function)

The ‘Timed Up and Go’ test (extremity and gait function)

Grip strength and physical fitness (six-minute walk test)

Mixed design ANOVA conducted on JASP.
