# Supplementary material for: Is walking netball an effective, acceptable and feasible method to increase physical activity and improve health in middle- to older age women?: A RE-AIM evaluation
Source: Int J Behav Nutr Phys Act. 2021 Oct 19;18:136. doi: 10.1186/s12966-021-01204-w (PMC8524399; doi:10.1186/s12966-021-01204-w)
Supplement: Supplementary file 3 — Additional file 3. Physical function measures record during the quasi-experimental study. [file 12966_2021_1204_MOESM3_ESM.docx]

**Additional File 3**

**Physical function measures record during the quasi-experimental study**

***Extremity and gait function***

The SPPB was used to examine extremity and gait function through markers of gait speed, mobility and static balance^1^. A 4-metre walk test assessed gait speed. Specifically, participants were asked to walk comfortably over 4-metres, where a short duration to complete the test represents greater function. A sit-stand test examined mobility, where participants were asked to stand from a chair without assistance. If this phase of the test was completed, participants were requested to complete a further five sit-stand movements in the quickest time possible. Static balance was measured through three hierarchal balance postures (normal stance, semi-tandem, tandem) (held for 10 seconds). Each component of the battery is scored (0 to 4) and calculated as a total score (0 to 12), with higher scores indicating greater extremity function. The SPPB has demonstrated an internal consistency score of .76 with older adults^1^.

Additionally, the TUG^2^ was used to understand functional movements. Following familiarization, the TUG required participants to stand from a standard chair’ (approximately 46 cm), walk at a safe and comfortable pace to a line on the floor three meters away, turn around and return to the chair and sit down again. Time is recorded for the duration of the test, with lower durations reflecting greater physical function. Times above 13.5 seconds are a clinical threshold for falls and frailty risk^2^. The TUG holds excellent intra- and inter-rater, and re-test reliability in healthy adults and primary care patients^3^.

***Muscular Strength***

Grip strength was measured with a hand grip dynameter (Takei; Digital 5401) and provided an indicator of muscular strength. Good evidence has indicated grip strength to be a predictor of overall muscular strength, physical function, frailty and health^4^. Following the standardized protocol^4^, the dynameter was adjusted to ensure the middle phalanx was securely gripping the inner handle. Whilst standing, participants held the dynameter in their dominant hand with the dominant arm parallel to the body and held their grip maximally. Measures were repeated three times with the greatest score used during analysis (measured in kg).

***Physical fitness***

Predictions of physical fitness were provided by the 6MWT^5^. Following instruction to cover the greatest distance possible at a normal walking pace (i.e., whilst able to talk and without running), participants walked between two lines (12-metres apart) for a period of six-minutes. During the test the researcher walked alongside the participant (to ensure compliance with the protocol and to time the test). During the test participants were permitted to stop and rest as required. Total distance was recorded, with greater distances reflecting greater functional physical fitness. The 6MWT is a valid and reliable sub-maximal fitness test for older adults where feasibility, frailty and pre-existing complications challenge the adoption of absolute objective testing^5^.

**References**

1. Guralnik JM, Simonsick EM, Ferrucci L, Glynn RJ, Berkman LF, Blazer DG, et al. A short physical performance battery assessing lower extremity function: association with self-reported disability and prediction of mortality and nursing home admission. J Ger, 1994;49:M85-M94.
2. Podsiadlo D, Richardson S. The timed “Up & Go”: a test of basic functional mobility for frail elderly persons. J Am Ger Soc. 1991;39:142-148.
3. Chan PP, Tou JIS, Mimi MT, Ng SS. Reliability and validity of the timed up and go test with a motor task in people with chronic stroke. Arch Phys Med Rehab. 2017;98:2213-2220; doi: 10.1016/j.apmr.2017.03.008.
4. Taekema DG, Gussekloo J, Maier AB, Westendorp RG, de Craen AJ. Handgrip strength as a predictor of functional, psychological and social health. A prospective population-based study among the oldest old. Age Age. 2010;39:331-337.
5. Harada ND, Chiu V, Stewart AL. Mobility-related function in older adults: assessment with a 6-minute walk test. Arch Phys Med Rehab, 1999;80:837-841.
