# Supplementary material for: Is walking netball an effective, acceptable and feasible method to increase physical activity and improve health in middle- to older age women?: A RE-AIM evaluation
Source: Int J Behav Nutr Phys Act. 2021 Oct 19;18:136. doi: 10.1186/s12966-021-01204-w (PMC8524399; doi:10.1186/s12966-021-01204-w)
Supplement: Supplementary file 4 — Additional file 4. Qualitative analysis. [file 12966_2021_1204_MOESM4_ESM.docx]

**Additional File 4**

**Qualitative analysis**

***Triangulated Thematic Analysis***

| **Theme** | **Sub-theme** | **Micro-theme** |
| --- | --- | --- |
| ***Effectiveness of participation*** | *Psychosocial health* |  |
|  | *Quality of life* |  |
|  | *Physical health* | Physical function |
|  |  | Physical fitness |
|  | *Mental health* | Mental health conditions |
|  |  | Cognition |
|  |  | Confidence |
|  | *The Women’s Institute* |  |
|  |  |  |
| ***Enablers for participation*** | *Logistics* |  |
|  | *The Women’s Institute* |  |
|  | *Social support* |  |
|  | *Function and movement* |  |
|  | *Love of netball* |  |
|  | *Internal motivation* |  |
|  | *Self-efficacy and confidence* |  |
|  |  |  |
| ***Barriers to participation*** | *Competition* |  |
|  | *Facilities* |  |
|  | *Others ability* |  |
|  | *Personal competence* |  |
|  | *Time* |  |
|  | *Injury* |  |
|  |  |  |
| ***Sustainability*** | *Funding* |  |
|  | *Multiple hosts* |  |
|  | *Organisation* | Tailored delivery |
|  |  | Regional adaptions |
|  |  | Funding |

***Triangulated Thematic Analysis continued***

| **Theme** | **Sub-theme** | **Micro-theme** |
| --- | --- | --- |
| ***Supporting basic needs*** | *Autonomy* |  |
|  | *Competence* |  |
|  | *Relatedness* |  |
|  |  |  |
| ***Factors which shape delivery*** | *Women’s Institute Host* |  |
|  | *Communication* |  |
|  | *The Women’s Institute* | Group structure |
|  |  | Involvement of the Women’s Institute group |
|  |  | Level of proactivity |
|  |  | Who plays? |
|  |  | Who hosts? |
|  | *Facilities* |  |
|  | *Host training* | Initial impressions |
|  |  | Improvements and adaptions |
|  |  | Structure of sessions |
|  |  | Effectiveness in practise |
|  |  | CDP and future development |
|  | *Coaching and support* |  |
|  | *Walking Netball sessions* | Structure |
|  |  | Multiple hosts |
|  |  | Adaption |
