# Supplementary material for: Is walking netball an effective, acceptable and feasible method to increase physical activity and improve health in middle- to older age women?: A RE-AIM evaluation
Source: Int J Behav Nutr Phys Act. 2021 Oct 19;18:136. doi: 10.1186/s12966-021-01204-w (PMC8524399; doi:10.1186/s12966-021-01204-w)
Supplement: Supplementary file 5 — Additional file 5. Quantitative analysis. [file 12966_2021_1204_MOESM5_ESM.docx]

**Additional File 5**

**Quantitative analysis**

***Multiple-level models***

Multilevel longitudinal growth models were used to analyse longitudinal data representing efficacy^1^. Multilevel growth models assess interindividual variability within intraindividual patterns of change (e.g., over time)^1^, and are useful for measuring equivocal data and challenges posed by multiple-baseline designs (e.g., missing data, dependent error structures, unequally spaced time points, heterogenous variance, moderation effects, non-normally distributed data and time-based covariance)^1,2^. Based upon previous effect sizes (*d*=.18 to .79^3,4^, five time-points and six-clusters we required 350 participants observe meaningful effects in our outcome measures^1^. Though, debate exists to what an acceptable sample size is within multilevel models^1^. Therefore, the sample size calculation was based upon the modelling of artificial data^5^. This indicated based on a small effect (*d*=.20), 1-β =.95, five time points and 1750 units of observation (each participant has five observations) 350 participants would be sufficient to observe a statistically meaningful effect in the data. Further, multilevel modelling offers increased statistical power to general linear modelling^1^.

Within each model, time points (Level 1) were nested into individual participants (Level 2). Study clusters (n=6) could have formed a third level, however there were insufficient higher level units (i.e., <15) for a robust analysis. The models were estimated using Iterative Generalised Least Squares and constructed in three stages. To establish the interclass correlation coefficient (ICC), a variance components (null) model was constructed. To understand period effects (i.e., where a change occurs), fixed predictors of time centred on their grand mean were entered into the random intercept model (model 1). To investigate interindividual variance; a random slope (model 2) was constructed through allowing outcomes to vary as a function of age and social economic status. At each stage model fit was calculated through 2*loglikelihood and χ2 distribution tests for significance. Missing data was addressed using the intention to treat principle^6^, where last observation carried forward and back was used to manage missing data completely at random. Data from participants with a pre-baseline and baseline value and at least one follow-up value (i.e., 3-, 6-, 12-months) were analysed.

Multilevel regression models were constructed to understand the interindividual variance in physical activity behaviour while accounting for variation across region as a function of multiple deprivation. Within these models, individual members (Level 1) were nested into their region (Level 2). WI groups could have formed a second and region a third level, however limited participants available to nest into groups eliminated this option. Age, partnership status, employment status, mental health and wellbeing, social isolation and quality of life outcomes were fixed predictors on the individual level. Multiple deprivation was a predictor of the random slope on the regional level. The models were estimated through Iterative Generalised Least Squares and constructed in three stages. To establish the ICC, a variance component only model was constructed. Following this, fixed predictors centred on their grand mean were entered into the random intercept model (model 1). To explore variance explained on the region-level; a random slope (model 2) was constructed through allowing physical activity to vary as a function of change in multiple deprivation across regions. Finally, level 1 interaction effects were tested between health outcomes and demographics. At each stage model fit was calculated through 2*loglikelihood and χ2 distribution tests for significance.

***Physical function***

Based on previous research^7^ reporting medium effects in markers of physical function a power calculation on G*Power (version 3.1.9.3) was conducted. Based upon a medium effect (*f* =.25), 1-β =.95, α =.05, two groups and two observations; 40 participants (20 per group) were required to observe a statistically meaningful effect in our measures of physical function. In addition, a 25% attrition rate was applied. Participants with missing T^1^ data were removed from analysis. Statistical significance was set at *p*=>.05 and the magnitude of change was represented by 95% confidence intervals. Descriptive statistics were calculated for all study variables with all data being normally distributed (skewedness and kurtosis ±1.96) and data not violating homogeneity of variance or covariance. A series of mixed-design (within-between) ANOVAs were used to assess the impact of the WN programme on physical function. Inspection of differences in confounding demographics at T^0^ was understood through independent samples t-tests. No meaningful differences were identified between the groups. Paired samples t-tests examined within samples effects post-hoc (with Cohen’s *d* reported). Data is represented as mean and standard deviation, and partial eta squared (*n^2^_p_*) was calculated as an effect size (small: *n^2^_p_* = .02, medium: *n^2^_p_* = .13, large: *n^2^_p_* = .26) for mixed-design ANOVAs.

**References**

1. Curren PJ, Obeidat K, Losardo D. Twelve frequently asked questions about growth curve modelling. J Cogn Dev, 2010;11:121-136; doi: 10.1080/1548371003699969.
2. Baek EK, Ferron JM. Multilevel models for multiple-baseline data: Modelling across-participant variation in autocorrelation and residual variance. Behav Res Methods, 2013;45:65-74; doi: 10.3758/s13428-012-0231-z.
3. Zubala A, MacGillivray S, Frost H, Kroll T, Skelton DA, Gavine A, et al. Promotion of physical activity interventions for community dwelling older adults: A systematic review of reviews. PloS one. 2017;12.
4. Pedersen MT, Vorup J, Nistrup A, Wikman JM, Alstrøm JM, Melcher PS, et al. Effect of team sports and resistance training on physical function, quality of life, and motivation in older adults. Scand J Med Sci Sports. 2017;27:852-864; doi: 10.1111/sms.12823.
5. Muthén BO, Curran P. General longitudinal modelling of individual differences in experimental designs: A latent variable framework for analysis and power estimation. Psych Methods. 1997;74:371-402.
6. McCoy CE. Understanding the intention-to-treat principle in randomized controlled trials. West J Emerg Med. 2017;18:1075.
7. Heyn P, Abreu BC, Ottenbacher KJ. The effects of exercise training on elderly persons with cognitive impairment and dementia: A meta-analysis. Arch Phys Med Rehab. 2004;85:1694-1704.
