# Supplementary material for: Is walking netball an effective, acceptable and feasible method to increase physical activity and improve health in middle- to older age women?: A RE-AIM evaluation
Source: Int J Behav Nutr Phys Act. 2021 Oct 19;18:136. doi: 10.1186/s12966-021-01204-w (PMC8524399; doi:10.1186/s12966-021-01204-w)
Supplement: Supplementary file 6 — Additional file 6. Findings: Multiple-baseline study. [file 12966_2021_1204_MOESM6_ESM.docx]

**Additional File 6**

**Findings: Multiple-baseline study**

***Participant demographics***

The mean age of participants (*n*=306) was 67.49±8.47 years. The majority of participants (99.3%) were white British, transitioning from full-time employment (89.5%) and married or in a partnership (81.4%). Most of the participants (74.6%) had a household income less than £40,000, 43.2% were degree educated, and 21.3% had played netball in past 12-months (measured pre-baseline). χ^2^ tests indicated a significant difference for ethnicity (χ^2^ = 284.05, *p* = 0.001), employment status (χ^2^ = 875.56, *p* = 0.001), marital status (χ^2^ = 787.44, *p* = 0.001), household income (χ^2^ = 237.18, *p* = 0.001) and education (χ^2^ = 102.37, *p* = 0.001).

***The effectiveness of Walking Netball on perceptions of mental health and wellbeing***

Mental health and wellbeing were above the midpoint and the ICC was .76. Data indicates participation in WN significantly improved mental health and wellbeing at 3-months (*b*=1.15, *p*=.001), 6-months (*b*=.96, *p*=.001) and 12-month (*b*=.87, *p*=.001) follow up (see Table 5). Meaningful variation exists between participants slopes (*b*=2.31, *p*=.001) over the course of the programme. Age did not significantly predict mental health and wellbeing, though social-economic status did (*b*=.71, *p*=.001).

***The effectiveness of Walking Netball on perceptions of loneliness***

The ICC was .83 and loneliness values were below the midpoint. WN contributed to significant reduced loneliness at 12-months (*b*=-.045, *p*=.001) only (see Table 5). There was meaningful variation between participants slopes (*b*=.008, *p*=.001). Age and social economic status did not predict social isolation.

***The effectiveness of Walking Netball on perceptions of quality of life***

Physical function was below the midpoint and the ICC was .63. Participation in WN contributed to significantly reduce risk to physical function at 3-months (*b*=-.10, *p*=.001) and 12-months (*b*=-.011, *p*=.001), but not at 6-months. Statistically meaningful variation existed between participant slopes (*b*=.04, *p*=.001), and social economic status significantly predicted risk to physical function (*b*=-.10, *p*=.001). Further, perceptions of risk to feelings were below the midpoint with an ICC of .70. Over time WN contributed to meaningful changes at 3-months only (*b*=-.098, *p*=.001). Significant interindividual slope variation existed (*b*=-.04, *p*=.001) and social economic status meaningful predicted risk (*b*=-.11, *p*=.001). Moreover, risk to social activities were considerably below the midpoint and the ICC was .64. WN participation contributes to a significant reduction at 6-months (*b*=.09, *p*=.001) only. Meaningful variation exists between participants (*b*=.02, *p*=.001). Pain likewise was below the midpoint within an ICC of .74. WN reduces risk at 3-months only (*b*=-08, *p*=.001), where significant variation exists between participants slopes (*b*=.04, *p*=.001). Social economic status significantly predicts pain (*b*=-.10, *p*=.001). Overall quality of life was reported as below the midpoint. The ICC was calculated as .78. Participation in WN reduces risk to quality of life at 3-months (*b*=-.05, *p*=.001). meaningful interindividual variation exists (*b*=.00, *p*=.001) and social economic status significantly predicts perceptions (*b*=-.07, *p*=.001). Finally, perceptions of ability to undertake daily activities, perceptions of changes in health, health status, and social support were maintained over the course of the programme (see Table 5).

***The effectiveness of Walking Netball on physical activity behaviour***

The ICC for physical activity measured in MET was .61. Participation in WN contributed to improvements in MET at 3-months (*b*=188.3, *p*=.001) and 12-months (*b*=298.6, *p*=.001), but not 6-months. Significant interindividual variation exists between members random slopes (*b*=226934.1, *p*=.001) (see Table 5).
