# Supplementary material for: Is walking netball an effective, acceptable and feasible method to increase physical activity and improve health in middle- to older age women?: A RE-AIM evaluation
Source: Int J Behav Nutr Phys Act. 2021 Oct 19;18:136. doi: 10.1186/s12966-021-01204-w (PMC8524399; doi:10.1186/s12966-021-01204-w)
Supplement: Supplementary file 7 — Additional file 7. Findings: Quasi-experimental study - Does participation in Walking Netball (WN) improve extremity function and gait Function. [file 12966_2021_1204_MOESM7_ESM.docx]

**Additional File 7**

**Findings: Quasi-experimental study - Does participation in Walking Netball (WN) improve extremity function and gait Function**

***Overall physical function***

Analysis indicates a meaningful improvement in SPPB scores (*p*=.001, *n^2^_p_*=.18) (see Table 6). However, analysis post-hoc identified non-meaningful differences. Indeed, post-hoc indicated a non-significant decrease of .36 (t=2.33, *p*=.15, *d*=.34) (95% CI -.79-.07) in the control group and a non-significant increase of .31 in the WN group (t=2.14, *p*=.22, *d*=.31) (95% CI -.09-.70). At baseline neither the intervention nor control group presented scores reflecting physical dysfunction.

***Gait speed***

Participation in the WN programme contributed to meaningful improvements in gait speed (*p*=.012, *n^2^_p_*=.13) when compared to the control group (see Table 6). Post-hoc paired samples t-tests demonstrated a meaningful reduction of -.57 seconds (t=5.98, *p*=.001, *d*=.86) (95% CI .309-.839) in the intervention group, while a non-significant reduction of .20 (t=1.939, *p*=.352, *d*=.28) (95% CI .085-.490) was observed in the control group (see Figure 1).

***Sit-Stand Ability***

Participation in WN resulted in meaningful changes in the ability to sit and stand from a chair 5-times (*p*=.001, *n^2^_p_*=.59) when compared to the control group (see Table 6). A meaningful reduction in time to complete the test of 2.48 seconds was observed in the WN group (t=7.51, *p*=.001, *d*=1.10) (95% CI 1.57-3.39). While an increase of 1.40 seconds was observed in the control group (t=7.51, *p*=.001, *d*=1.10) (95% CI 1.57-3.39) (see Figure 2).

***Balance***

Participation in the WN programme contributed to small but meaningful improvements in balance scores (*p*=.009, *n^2^_p_*=.04) when compared to the control group (see Table 6). The WN group improved significantly by .27 points (t=2.68, *p*=.06, *d*=.39) (95% CI -.07-.55), while a non-significant decrease of .05 was observed in the control group (t=.42, *p*=1.00, *d*=.06) (95% CI -.35-.26).

***Functional Ability***

Participation in WN contributed to improvements in functional ability as measured through the TUG (*p*=.012, *n^2^_p_*=.13) when compared to the control group (see Table 6). Post-hoc paired samples t-tests demonstrated a meaningful reduction of .53 seconds (t=3.92, *p*=.001, *d*=.57) (95% CI -.90--.16) in the intervention group and a significant increase of .37 seconds (t=2.57, *p*=.008, *d*=.37) (95% CI -.03-.78) was observed in the control group (see Figure 3).

**Does Participation in Walking Netball Improve Muscular Strength**

Participation in the WN group did not contribute to meaningful adaptions in grip strength (*p*=.87, *n^2^_p_*=.00). Upon eliminating the group interaction effect, there were small observable differences between time points (*p*=.001, *n^2^_p_*=.20) and groups (*p*=.01, *n^2^_p_*=.14)

**Does Participation in Walking Netball Improve Physical Fitness**

Participation in WN contributed to meaningful differences in physical fitness (measured with the 6MWT) (*p*=.001, *n^2^_p_*=.63) when compared to the control group (see Table 6). Post-hoc analysis indicates meaningful differences in both groups. More specifically, the control group improved significantly by 22.01 meters (t=3.59, *p*=.001, *d*=.50) (95% CI 5.17-38.85), while the WN group improved to a greater extent by 52.26 meters (t=9.96, *p*=.001, *d*=1.38) (95% CI 37.84-66.68) (see Figure 4).
